# Comparative analysis of cadmium uptake and distribution in contrasting canadian flax cultivars

**DOI:** 10.1186/s13104-020-05265-1

**Published:** 2020-09-07

**Authors:** Megan A. House, Lester W. Young, Xia Liu, Karsten Liber, Axel Diederichsen, Helen M. Booker

**Affiliations:** 1 Department of Plant Sciences, University of Saskatchewan, 51 Campus Drive, Saskatoon, SK S7N 5A8 Canada; 2 Toxicology Centre, University of Saskatchewan, Saskatoon, SK Canada; 3 Saskatoon Research and Development Centre, Plant Gene Resources of Canada, Agriculture and Agri-Food Canada, 107 Science Place, Saskatoon, SK S7N 0X2 Canada; 4 Department of Plant Agriculture, Ontario Agricultural College, University of Guelph, 50 Stone Rd E, Guelph, ON N1G 2W1 Canada

**Keywords:** Flax, Breeding, Cadmium, Heavy metal, Development, Uptake, Translocation

## Abstract

**Objective:**

Humans consume low quantities of cadmium (Cd), a non-nutritive and potentially toxic heavy metal, primarily via the dietary intake of grains. A trial experiment was conducted to investigate physiological and developmental differences in Cd content in four flax cultivars (‘AC Emerson’, ‘Flanders’, ‘CDC Bethune’, and ‘AC McDuff’) as part of a study to provide information that will assist in the breeding of low Cd-accumulating flax cultivars. Our objective was to identify varietal differences in the uptake and distribution of Cd in various tissues among flax cultivars grown in naturally Cd-containing soil in a controlled environment.

**Results:**

Cadmium concentration was dependent on genotype, developmental stage, and tissue type, as well as their interaction. Cadmium concentration was higher in roots and leaves, relative to all other tissues, with a general trend of decreasing Cd content over time within leaves and stems. Notably, the concentration of Cd was higher in ‘AC Emerson’ relative to ‘AC McDuff’ across tissues and ages, including the seeds, while the concentration of ‘Flanders’ was higher than in ‘AC McDuff’ in seeds and other reproductive organs but similar in roots and leaves. The results suggest varietal differences in the mechanisms that determine Cd content in seeds.

## Introduction

Cadmium (Cd), a heavy, non-nutritive, and potentially toxic metal, is found naturally in the environment at low levels, although anthropogenic activities have resulted in substantially higher levels in the soil [1]. Soil-borne Cd is not a direct concern for human health, however, food-borne Cd is the major route of exposure for most people [2]. Cadmium is readily taken up by plants through their roots and some crops may accumulate high levels of Cd in their seeds [3, 4].

There are currently no recommendations for acceptable levels of Cd in flaxseed, however, the recommended weekly maximum dietary intake set out by the European Food Safety Authority is 2.5 µg of Cd per kg of body weight [5]. The concentration of Cd in flaxseed may influence food processor and consumer choices, particularly in the health food sector. To improve marketability and healthfulness of Canadian flaxseed, breeding of Canadian flax cultivars will include selection for low Cd-accumulating genotypes.

The efficiency with which Cd uptake occurs, and where it is distributed and accumulated within plants, is determined partially by genetics [4, 6–9]. Variety-specific differences in the concentration of Cd accumulated in flax seeds have been studied but these studies did not investigate differences in uptake and translocation [10–13]. Identifying developmental and/or tissue-specific differences in Cd concentration will increase our understanding of genetic factors associated with low-Cd flaxseed and allow for more strategic and accelerated breeding approaches.

This experiment was performed as part of a larger investigation of seed Cd concentrations in different flax germplasm resources for the purposes of breeding low-Cd accumulating cultivars. This study investigates the genotypic differences in Cd concentration between vegetative tissues and reproductive structures throughout development. The results of this investigation will be used for designing experiments to identify genes that regulate Cd accumulation in flaxseed.

## Main text

### Methodology

Four Canadian flax cultivars were selected for this study: ‘CDC Bethune’ [14], ‘AC Emerson’ [15], ‘Flanders’ [16], and ‘AC McDuff’ [17]. Seed was from the Crop Development Centre’s flax breeding program, where they are used as controls. The materials and corresponding voucher specimens are available at Plant Gene Resources of Canada (Saskatoon, SK, Canada), under the conditions of the Multilateral System for Access and Benefit-sharing of the International Treaty on Plant Genetic Resources for Food and Agriculture. Plants were seeded into 4 L pots and grown in a controlled environment growth chamber (see Additional file 1 for details on soil, watering, fertilizing, and pest control).

Four developmental stages were selected for tissue collection based on morphological markers (first bud stage, first flower stage, full flowering stage and maturity) (Additional file 1). Tissues were collected (roots, stems, leaves, shoot tips, flowers, immature bolls, and seeds) at all appropriate stages, and were stored at -80 °C. Tissues were freeze-dried and ground into a powder using a 2010 Geno/grinder (SPEX CertiPrep, Inc., Methucen, NJ). Ground, freeze-dried tissue samples were processed at the Toxicology Centre, University of Saskatchewan, before Cd quantification using inductively coupled plasma-mass spectrometry (ICP-MS).

All statistical analyses were performed in RStudio, version 3.6.3 [18]. Prior to performing analyses of variance (ANOVA), we confirmed the data had equal variance and was normally distributed. Two-way ANOVA was used to test the effects of genotype and tissue on Cd accumulation in reproductive structures using the core R stats package function, aov() [18]. A three-way mixed ANOVA was performed using the rstatix package [19] function, anova_test(), to test the effects of genotype, age, tissue on Cd accumulation in vegetative tissues, followed by simple two-way ANOVA when appropriate. Tukey’s HSD was used to make pairwise comparisons and the level of significance accepted for all tests was p < 0.05.

Additional details on the materials and methods used are in Additional file 1.

### Results

#### *Accumulation of Cd in flowers, immature bolls and seeds*

**Fig. 1:**
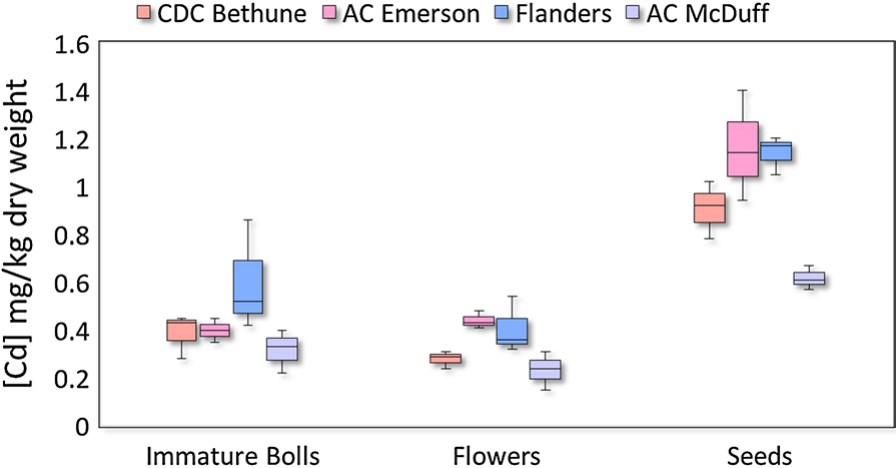
Boxplot diagram of Cd concentration in flax reproductive structures. Boxes represent the average Cd concentration of three replicates for each genotype/tissue combination

Using a two-way ANOVA we determined that genotype and tissue stably affect Cd concentration within reproductive structures (Additional file 2: Table S1). Specifically, seeds had a higher concentration of Cd than flowers and immature bolls, and there was no difference between flowers and bolls (Fig. 1). The differences in Cd concentration between seeds and flowers, and seeds and bolls, were stable, with the concentration in ‘AC McDuff’ lower than that in ‘AC Emerson’ and ‘Flanders’, but the same as that in ‘CDC Bethune’ (Fig. 1).

#### *Genotype, tissue anddevelopmentalstage interact to determine Cd concentration in flax*

Cd concentration was measured in roots, stems, leaves and shoot tips in the four flax cultivars at various stages of development. The three-way interaction among genotype, tissue, and age was significant (F_9,48_ = 4.383, p < 0.001), so statistical differences within tissues and ages were tested to understand the relationship between genotype and age, and genotype and tissue.

#### *Effect of genotype and age on Cd concentration in roots, stems, leaves and shoot tips*

**Fig. 2:**
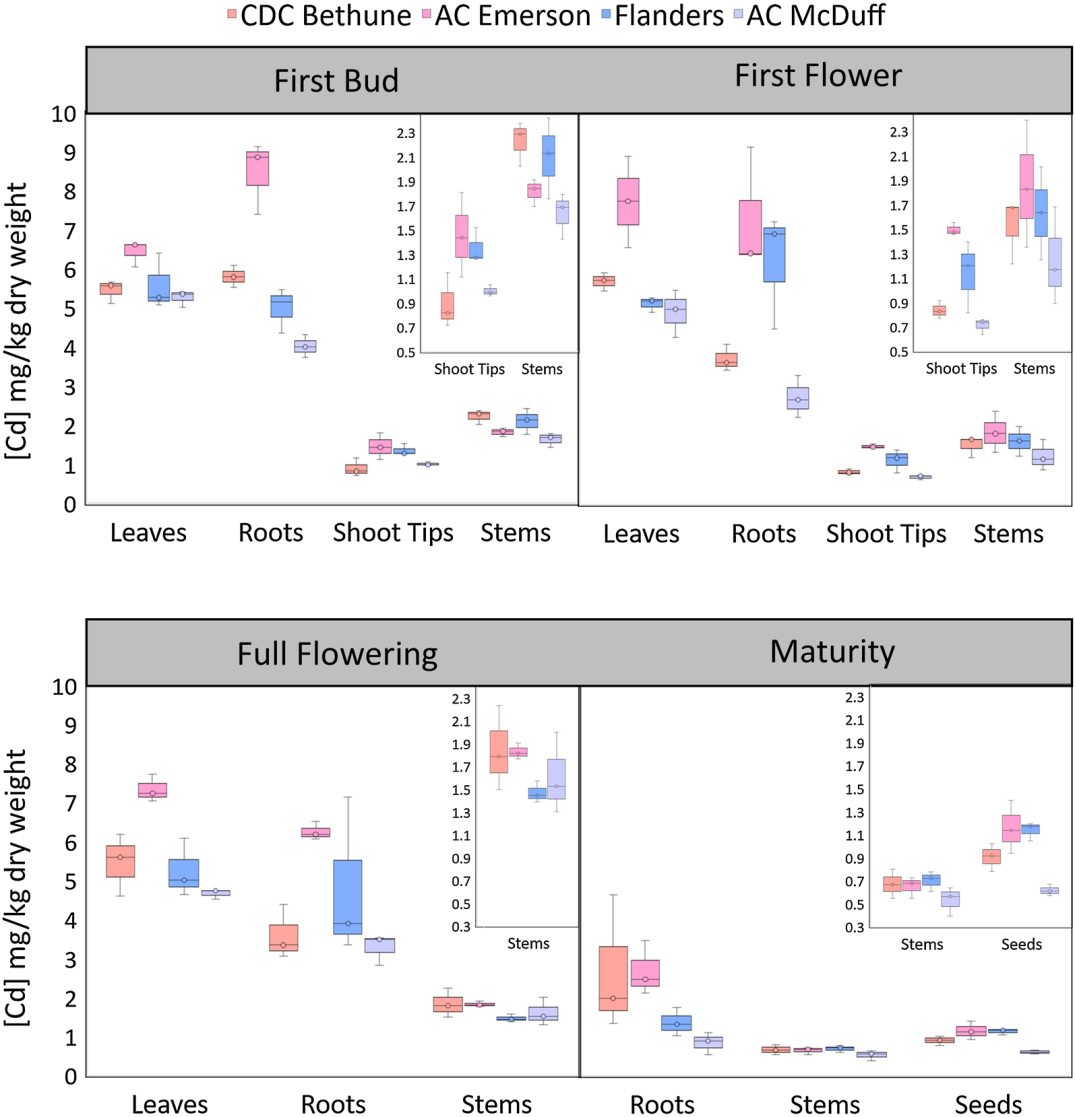
Boxplot diagram of Cd concentration in vegetative tissues throughout development. Boxes represent the average Cd concentration of three replicates for each genotype/tissue combination for the first bud, first flower, full flowering, and maturity stages of development

Within leaves and shoot tips, Cd concentration was stably affected by genotype (Fig. 2; Additional file 2: Table S2, Additional file 3). The concentration of Cd in leaves was similar in ‘CDC Bethune’, ‘Flanders’ and ‘AC McDuff’ (mean of 5.27 ± 0.10 mg/kg), and was significantly lower than in ‘AC Emerson’ (7.18 ± 0.29 mg/kg). The level of Cd in shoot tips between ‘AC Emerson’ and ‘Flanders’ was similar, and both were higher than in ‘CDC Bethune’ and ‘AC McDuff’.

In stems, stable effects of both genotype and age on Cd concentration were observed, though the effect of genotype was less significant (Additional file 2: Table S2, Additional file 3). Concentrations ranged from 0.39 to 2.42 mg/kg and the effect of genotype was driven by a difference between ‘AC McDuff’ (1.26 ± 0.15) and ‘CDC Bethune’ (1.57 ± 0.18) (Fig. 2; Additional file 3). Similarly, in stems of all genotypes, there was an overall pattern of decreasing Cd concentration with increasing age.

Within roots, there was a significant genotype-by-age interaction (Fig. 2; Additional file 2: Table S2, Additional file 3). The developmental pattern of Cd concentration was similar in ‘AC Emerson’ and ‘Flanders’, and in ‘CDC Bethune’ and ‘AC McDuff’. ‘AC Emerson’ and ‘Flanders’ exhibited stable Cd concentrations through the first three stages of development, which declined significantly at maturity. Roots of ‘CDC Bethune’ and ‘AC McDuff’, however, had a higher concentration of Cd at the first developmental stage compared to maturity, but showed no differences among the first flower stage, full flowering stage, and at maturity (Fig. 2; Additional file 3). Genotypic differences were not observed in roots at maturity, however, most interesting is the consistently higher Cd concentration in ‘AC Emerson’ compared to ‘AC McDuff’ during all stages of active growth.

#### *Effect of genotype *and* tissue on Cd concentration through development*

Within developmental stages, the effects of genotype, tissue, and the genotype-by-tissue interaction were compared using a two-way mixed ANOVA. Differences between tissues were dependent on genotype during the first three stages of development only. At maturity, only roots and stems were collected, with roots having a stably higher Cd concentration than stems (Fig. 2; Additional file 2: Table S3). Notably, the concentration of Cd in leaves was consistently higher than that in stems and shoot tips for all genotypes. The concentration in roots and leaves were similar, but some genotype-specific differences were observed at the first three developmental stages (Fig. 2).

### Discussion

In Canada, flax is grown predominantly in the Prairie province of Saskatchewan, but also in Alberta and Manitoba [20]. Cd concentrations in Saskatchewan soils from the AP soil horizon (that is, in the upper layer soil that has been disrupted by human activity) range from 0.127 mg/kg to 0.456 mg/kg, with an average concentration of 0.32 mg/kg [21]. In other parts of the Prairies, soils contain anywhere from 0.1 – 7.9 mg/kg of Cd, but the vast majority contain less than the worldwide average of 0.53 mg/kg [22]. The soil used in our study contained ~ 0.45 mg/kg of Cd and can therefore be considered representative of most agricultural sites in the Canadian Prairies.

As expected, genotype has a significant effect on Cd concentration in reproductive structures and we determined ‘AC Emerson’ and ‘Flanders’ are consistently higher accumulators than ‘AC McDuff’. A previous study also found that ‘Flanders’ accumulates more Cd in its seeds than ‘AC McDuff’ [13], and our study indicates this relationship is not restricted to the seed. In addition, we found that the concentration of Cd is higher in the seeds than in flowers and immature bolls, which indicates that a substantial proportion of seed Cd is specifically redistributed to the seeds during the seed-filling stage.

The Cd concentration in seed in our study ranged from 0.57 mg/kg in ‘AC McDuff’ to 1.40 mg/kg in ‘AC Emerson’. A quick calculation shows that, even using the lowest accumulator included in this study, it would not be difficult to exceed the weekly-recommended 2.5 µg Cd per kg of body weight per week after consuming flaxseed. For example, according to this recommendation, a person weighing 60 kg (~ 132 lbs) should consume < 150 µg of Cd/week. The health claim for whole flaxseed states that the daily amount of ground flax seed to reduce cholesterol is 40 g (0.28 kg/week) [23]. If the seed contains 0.57 mg/kg (i.e. the concentration in ‘AC McDuff’), an individual following the health claim could be inadvertently ingesting 160 µg of Cd per week from flaxseed alone. This rough calculation puts into perspective the need to effectively and efficiently breed low Cd-accumulating cultivars.

The high level of Cd in ‘AC Emerson’ relative to ‘AC McDuff’ was consistent in most tissues. Had these two cultivars been compared in isolation, one would likely conclude that in flax the differences in seed Cd accumulation are due solely to initial differences in Cd uptake by the roots. While initial differences in Cd uptake by the roots appear to contribute to varietal differences, the story is more complicated when additional varieties are considered. ‘Flanders’, for example, also contained more Cd in its seeds than ‘AC McDuff’, but approximately the same concentration in roots and leaves. This is an important finding because it indicates that the concentration of Cd in seeds may also occur because of genotypic differences in the redistribution of Cd to the seeds during maturity, rather than solely from initial differences in Cd uptake.

It is important to note that ‘AC Emerson’ may represent a special case of high Cd accumulation since it is also tolerant to iron-deficiency chlorosis [15]. Iron transporters in rice and Arabidopsis have been reported to also transport Cd [24–26]. *NRAMP1*, specifically, is more highly expressed when plants are growing in iron-depleted soils [25]. Greater iron-use efficiency in ‘AC Emerson’ under low iron conditions could result in a concordant increase in Cd uptake. The consistently high levels of Cd observed in ‘AC Emerson’ may represent a unique difference in abundance and/or expression of root iron transporters. Mobilization and transport of Cd during reproductive development may be similar in ‘AC Emerson’ compared to the other varieties and it may be more typical that the genotypic differences in seed Cd accumulation are due to differences in redistribution of Cd from aerial tissues to the seeds.

#### Conclusion

Overall, results of our study suggest that genotypic differences in Cd accumulation in flax seeds may be partly due to differences in the redistribution of Cd to the seeds during maturation. In the case of chlorosis-tolerant ‘AC Emerson’, greater initial Cd uptake may contribute to differences in seed Cd levels. These results will be useful for future projects aimed at understanding the molecular mechanisms that determine varietal differences in the accumulation of Cd in flax seed.

## Limitations

This study had one main limitation. Health and safety requirements for available growth facilities prohibited the application of Cd to soil or any other media. We were able to use Cd-containing soil collected from a field site but were unable to include a Cd-free control. This limited our study because we were unable to determine if the Cd taken up by plants had an effect on their growth or if the varietal differences in Cd concentration within tissues and over ages are affected by the original soil Cd concentration.

## Supplementary information

**Additional file 1.** Additional details pertaining to materials and methods for soil preparation, seeding and growth conditions, watering, tissue collection, Cd quantification and statistical analyses.

**Additional file 2: Tables S1–S3.** Results of the 1) two-way ANOVA to assess the effect of genotype, tissue, and the genotype-by-tissue interaction on Cd concentration in reproductive structures, 2) two-way ANOVA to assess the effect of genotype, age, and the genotype-by-age interaction in Cd concentration within vegetative tissues, and 3) two-way mixed ANOVA to assess the effect of genotype, tissue, and the genotype-by-tissue interaction on Cd concentration within developmental stages.

**Additional file 3: Figure S1.** Boxplot diagram presenting Cd concentration (mg/kg dry weight) for each cultivar expressed specifically within vegetative tissues.

## Data Availability

The datasets used and/or analysed during the current study are available from the corresponding author on reasonable request.

## Abbreviations

ANOVA: Analysis of Variance
Cd: Cadmium
SK: Saskatchewan
