# Supplementary material for: Comparative analysis of cadmium uptake and distribution in contrasting canadian flax cultivars
Source: BMC Res Notes. 2020 Sep 7;13:424. doi: 10.1186/s13104-020-05265-1 (PMC7487502; doi:10.1186/s13104-020-05265-1)
Supplement: Supplementary file 1 — Additional file 1. Additional details pertaining to materials and methods for soil preparation, seeding and growth conditions, watering, tissue collection, Cd quantification and statistical analyses. [file 13104_2020_5265_MOESM1_ESM.docx]

**Additional Details for Materials and Methods**

**Methodology**

***Soil Preparation***

Surface layer soil was collected using shovels to a depth of 10 centimeters from the Agriculture and Agri-Food Canada field site (Saskatoon, SK) in September 2018. The soil is a Dark Brown Chernozem. The rotation includes cereals and rapeseed using standard fertilizer and a low till system. The soil was air dried for several months and was then mixed thoroughly with a soil mixer at the University of Saskatchewan Phytotron facility. Mixed soil was filtered through a 2 mm sieve and stored in large, plastic totes for future use. The Cd concentration of the soil was tested prior to the experiment by ICP-MS (details below) and was found to be 0.45 mg/kg.

***Plant Material, Seeding and Growth Conditions***

Four Canadian flax varieties were selected for this study: ‘CDC Bethune’ (24), ‘AC Emerson’ (20), ‘Flanders’ (25), and ‘AC McDuff’ (26). ‘Flanders’ and ‘AC McDuff’ were selected because previously published research indicates they accumulate high and low levels of Cd in the seeds, respectively (15). Seed was from the Crop Development Centre’s flax breeding program (University of Saskatchewan, Saskatoon, SK, Canada), for which they are used as controls. The material and corresponding voucher specimens are available at Plant Gene Resources of Canada in Saskatoon, SK, Canada, under the conditions of the Multilateral System for Access and Benefit-sharing of the International Treaty on Plant Genetic Resources for Food and Agriculture. Plants were seeded into 4 L pots containing 1300 mL of dry, mixed soil (soil described above). To aid in efficient germination, an additional 500 mL of 50:50 outdoor soil : SunGro® Horticulture potting mix (Seba Beach, Alberta) was added to the top of each pot. In our experiment, each pot, containing 4 plants, was considered a biological sample where tissues from all 4 plants were combined to gain adequate tissue for Cd analysis. Position of pots within the growth chamber was completely randomized and pots were rotated within the chamber on a weekly basis. Pots were fertilized weekly with 200 ml of 15–30-15 supplemented with 0.1 g.L^− 1^ of Cu_2_SO_4_.5H_2_O, and were located in controlled environment growth chambers (18 h / 6 h, 22°/17 °C day/night cycle) at the University of Saskatchewan. To minimize infestation by thrips, *Hypoaspis miles* was applied to each pot upon germination, and cucumeris (*Amblyseius****c****ucumeris*) was applied to each pot throughout the experiment as needed.

***Watering***

All pots were watered based on water holding capacity (WHC), which was determined by gravimetric analysis. The percentage of the WHC used for watering varied throughout the development of the plants as water demands fluctuated, but was consistent across pots at any given time.

***Tissue Collection***

Tissues and reproductive structures harvested for all samples were the combination of those collected from 4 plants of the same genotype that were grown in the same pot and harvested at the same time. Four developmental stages were selected for tissue collection and these were based on morphological markers that could ensure consistency in tissue collection between diverse genotypes. Tissues were collected based on the following markers: (1) the appearance of the first visible flower bud, (2) the appearance of the first open flower, where petals are open and the male and female reproductive organs are visible, (3) full flowering, where there is extensive branching, the majority of flower buds have opened to reveal flowers, and there are immature bolls/capsules forming and (4) maturity, when >80% of the bolls/capsules contain brown seeds that rattle. When the first bud and first flower were visible, we collected tissue samples from the roots, stems, leaves, and shoot tips. At full flowering, tissue was collected from roots, stems, leaves, flowers, and immature (green) bolls. At physiological maturity, tissue was collected from roots, stems, and seeds. Root tissue was collected by rinsing the roots from a single pot with water to gently remove soil and extraneous organic matter, placing all root tissue in a plastic tube and storing at -80 °C prior to grinding. Leaf samples were the combination of the oldest 20 leaves from the 4 plants/pot for each genotype. Stems corresponding to these sections of leaves were harvested and combined as a single leaf sample. Shoot tip samples at the first bud and first flower stage were comprised of the top 3 cm of the main stem from all 4 plants/pot for each genotype. Aerial tissues were collected in capped 50 ml polypropylene tubes and stored at -80 °C prior to freeze drying and grinding. Flower, immature boll and seed samples consisted of whole structures and were stored at -80 °C following their collection. All tissues were collected and stored in capped 50 ml polypropylene tubes.

***Cd Quantification***

All tissues were freeze-dried and ground into a powder in the 50 ml storage tubes. Homogenization was performed for 1300 rpm for 60 sec using a 2010 Geno/grinder (SPEX CertiPrep, Inc., Methucen, NJ) and 2-4 8 mm diameter ceramic grinding media. Stem and root tissue required multiple runs for complete homogenization. Ground tissue samples (approx. 5-10 g per) were subsampled into 5 ml transport tubes and further processed at the Toxicology Centre, University of Saskatchewan before Cd quantification using inductively coupled plasma-mass spectrometry (ICP-MS).

Dried subsamples of plant tissue were digested using a MARS 5 microwave digestion system (CEM Corporation, Matthews, NC, USA) equipped with closed Teflon vessels. About 0.1 g of dried tissues was weighed and added into each vessel, followed by addition of 2.0 ml of 69% Nitric Acid (HNO_3_) (Optima Grade, Fisher Scientific, Toronto, ON) and 1.0 ml of 30% hydrogen peroxide (H_2_O_2_) (Ultra-Trace Analysis grade, Sigma-Aldrich, Oakville, ON). The vessels were capped tightly and placed in a fume hood for 30 min to initiate digestion of organic matter, and subsequently placed in the MARS system for microwave digestion. After complete digestion, the solution in each vessel was carefully transferred to a 30-ml high density polyethylene (Nalgene^®^) bottle and diluted with 5.0 ml ultrapure (MilliQ) water. Approximately 1.0 g of the resulting solution was filtered and further diluted by adding 4.5 g of MilliQ water. After this dilution, the final solution was in a 2% nitric acid matrix and ready for analysis via ICP-MS. Reagent blanks (containing only digestion reagents) and certified reference materials (CRM; spinach leaves, NRC-CNRC, Ottawa, ON, Canada) were run with the tissue samples at a frequency equaling 10% of the total samples.

The concentrations of Cd in the tissue samples were analyzed using an Agilent Technologies 8800 ICP-MS Triple Quadrupole spectrometer (Agilent, Santa Clara, CA, USA). Before sample analysis, the instrument performance was optimized using a standard tuning solution containing 1 µg/L each of Li, Y, Ce, Tl and Co in a matrix of 2% HNO_3_. An internal standard solution was added automatically online to all blanks, external standards and samples. The internal standard recoveries were always within 80-120% of nominal values. A standard calibration curve for Cd quantification was established using Cd concentrations of 0, 0.1, 1, 10, 100, and 500 µg/L. The correlation coefficient for each standard curve was always greater than or equal to 0.9999.

A natural water standard reference material (1640a; National Institute of Standards and Technology, Gaithersburg, MD, USA) was used as the instrument quality control sample (IQCS) to verify instrument performance and analytical accuracy. The spinach leaves were used as method quality control samples (MQCS) for documenting the efficiency of Cd extraction via microwave digestion and other sample preparation procedures. The mean value of IQCS recovery was within ±10% of the certified value, while the MQCS were within 80-120% of the certified value. Actual flax tissue sample extracts were only quantified after all QA/QC check had passed. Duplicate checks were run for every 10 samples to verify the reproducibility and stability of the instrument performance. Instrument data were collected using Agilent’s Masshunter^®^ software and processed in Excel for determination of Cd concentrations in each of the tissue samples.

***Statistical Analysis***

All statistical analyses were performed in RStudio, version 3.6.3 (27).

Prior to performing analyses of variance (ANOVA) on any group of data, we first confirmed that the data met the necessary assumptions. We used the rstatix package (28) in Rstudio to test the assumption of equal variance with Levene’s Test (levene_test()), and tested the assumption of normality using the shapiro_test() function to perform Shaprio’s.

**Effect of genotype and tissue on Cd concentration in reproductive structures**

For data collected from reproductive structures (flowers, immature bolls, and seeds), a two-way ANOVA was used to test the effects of genotype and tissue on Cd accumulation using the core R stats package function, aov() (27). Tukey’s HSD test was used to compare pairwise relationships between genotypes and between structures. The level of significance accepted for all tests was p<0.05.

**Effect of genotype, age, and tissue on Cd concentration in vegetative tissues**

A three-way mixed ANOVA was performed using the rstatix package (28) function, anova_test(), to test the effects of genotype, age, tissue on Cd accumulation. When the within-subject factor, tissue, violated assumptions of sphericity, the anova_test() function automatically applied a Greenhouse-Geisser correction. Following a significant three-way interaction, a two-way ANOVA was used to test the effects of genotype and age, within tissues, performed a two-way mixed ANOVA to test the effects of genotype and tissue within developmental stages. Tukey’s HSD was used to make pairwise comparisons. The level of significance accepted for all tests was p<0.05.
