# Supplementary material for: Comparative analysis of cadmium uptake and distribution in contrasting canadian flax cultivars
Source: BMC Res Notes. 2020 Sep 7;13:424. doi: 10.1186/s13104-020-05265-1 (PMC7487502; doi:10.1186/s13104-020-05265-1)
Supplement: Supplementary file 2 — Additional file 2: Tables S1–S3. Results of the 1) two-way ANOVA to assess the effect of genotype, tissue, and the genotype-by-tissue interaction on Cd concentration in reproductive structures, 2) two-way ANOVA to assess the effect of genotype, age, and the genotype-by-age interaction in Cd concentration within vegetative tissues, and 3) two-way mixed ANOVA to assess the effect of genotype, tissue, and the genotype-by-tissue interaction on Cd concentration within developmental stages. [file 13104_2020_5265_MOESM2_ESM.docx]

**Additional file 2. Table S1.** Two-way ANOVA to assess the effect of genotype, tissue, and the genotype-by-tissue interaction on Cd concentration in reproductive structures.

| **Effect** | **df** | **F** | **P-level** |
| --- | --- | --- | --- |
| Genotype | 3, 24 | 13.781 | **<0.0001** |
| Tissue | 2, 24 | 94.090 | **<0.0001** |
| Genotype x Tissue | 6, 24 | 2.477 | 0.0523 |

Significant effects are indicated in bold.

**Table S2.** Two-way ANOVA to assess the effect of genotype, age, and the genotype-by-age interaction in Cd concentration within vegetative tissues.

| **Tissue** | **Effect** | **df** | **F** | **P-level** |
| --- | --- | --- | --- | --- |
| Leaves | Genotype | 3, 24 | 26.4 | **<0.0001** |
|  | Age | 2, 24 | 0.578 | 0.569 |
|  | Genotype x Age | 6, 24 | 1.72 | 0.158 |
| Shoot Tips | Genotype | 3, 16 | 14.9 | **<0.0001** |
|  | Age | 1, 16 | 2.74 | 0.117 |
|  | Genotype x Age | 3, 16 | 0.91 | 0.458 |
| Stems | Genotype | 3, 32 | 3.25 | **0.0340** |
|  | Age | 3, 32 | 53.3 | **<0.0001** |
|  | Genotype x Age | 9, 32 | 1.31 | 0.272 |
| Roots | Genotype | 3, 32 | 26.0 | **<0.0001** |
|  | Age | 3, 32 | 36.5 | **<0.0001** |
|  | Genotype x Age | 9, 32 | 2.8 | **0.0150** |

Significant effects are indicated in bold.

**Table S3.** Two-way mixed ANOVA to assess the effect of genotype, tissue, and the genotype-by-tissue interaction on Cd concentration within developmental stages.

| **Tissue** | **Effect** | **df** | **F** | **P-level** |
| --- | --- | --- | --- | --- |
| First Bud | Genotype | 3, 8 | 15.8 | **0.0010** |
|  | Tissue | 3, 24 | 705.0 | **<0.0001** |
|  | Genotype x Tissue | 9, 24 | 23.3 | **<0.0001** |
| First Flower | Genotype | 3, 8 | 14.8 | **0.0010** |
|  | Tissue* | 1.6, 12.8 | 175.0 | **<0.0001** |
|  | Genotype x Tissue* | 4.81, 12.8 | 7.01 | **0.0200** |
| Full Flowering | Genotype | 3, 8 | 11.1 | **0.0030** |
|  | Tissue | 2, 16 | 95.4 | **<0.0001** |
|  | Genotype x Tissue | 6, 16 | 3.06 | **0.0340** |
| Maturity | Genotype | 3, 8 | 2.77 | 0.111 |
|  | Tissue | 1, 8 | 21.6 | **0.0020** |
|  | Genotype x Tissue | 3, 8 | 2.73 | 0.114 |

Significant effects are indicated in bold.

* Degrees of freedom correction applied because of the sphericity assumption violation.
