# Supplementary material for: Comparative analysis of cadmium uptake and distribution in contrasting canadian flax cultivars
Source: BMC Res Notes. 2020 Sep 7;13:424. doi: 10.1186/s13104-020-05265-1 (PMC7487502; doi:10.1186/s13104-020-05265-1)
Supplement: Supplementary file 3 — Additional file 3: Figure S1. Boxplot diagram presenting Cd concentration (mg/kg dry weight) for each cultivar expressed specifically within vegetative tissues. [file 13104_2020_5265_MOESM3_ESM.docx]

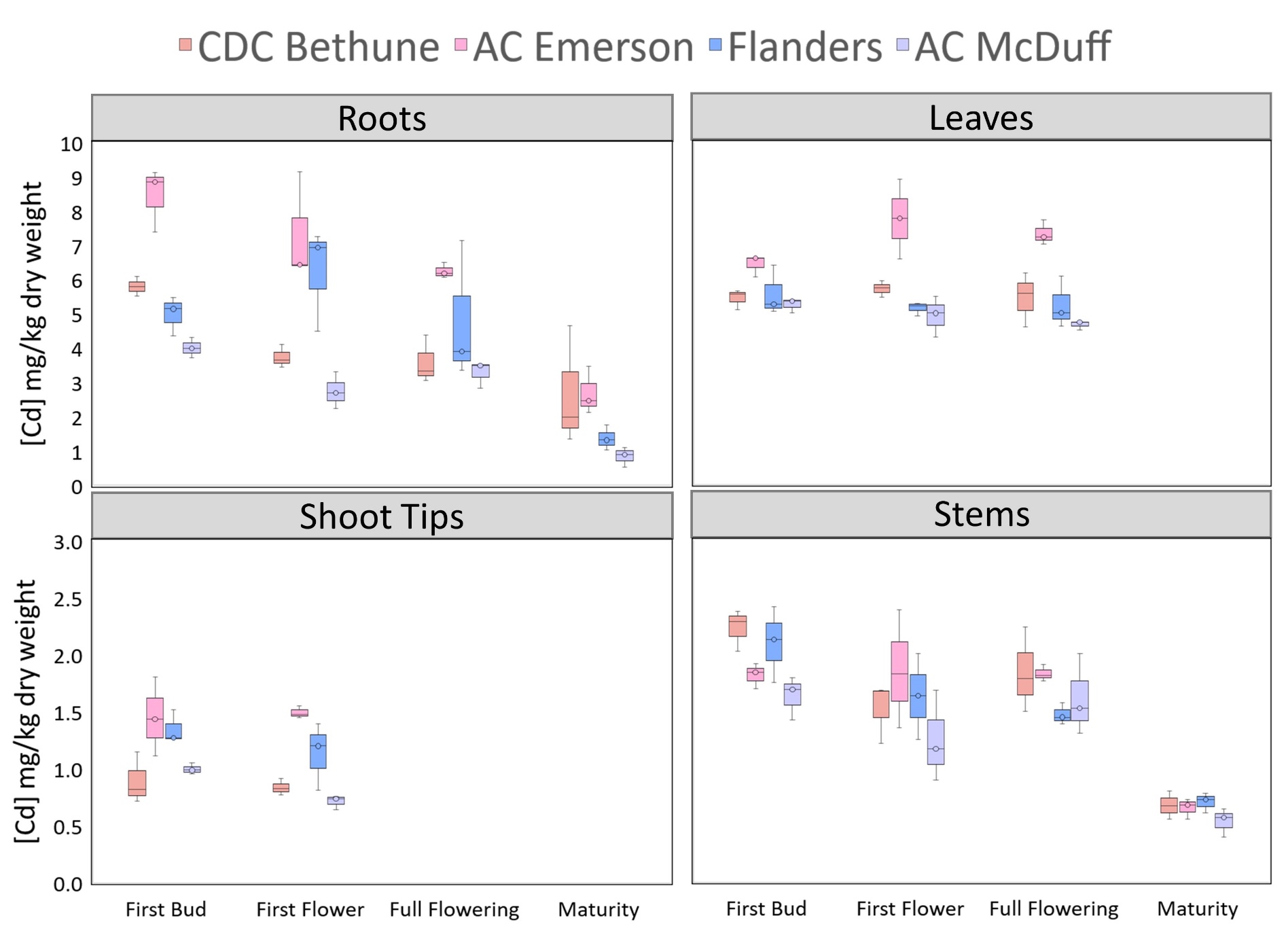


Additional file 3. Figure S1. Boxplot diagram of Cd concentration for cultivars within each vegetative tissue and throughout development. All boxes were calculated using the average Cd concentrations from three biological replicates. To fully demonstrate the genotypic differences between cultivars in shoot tip and stem tissues, the Cd concentration (y axis) is expressed on a smaller scale than for root and leaf tissues.
